# KCNA1 promotes the growth and invasion of glioblastoma cells through ferroptosis inhibition via upregulating SLC7A11

**DOI:** 10.1186/s12935-023-03199-9

**Published:** 2024-01-03

**Authors:** Weichao Wang, Yang Zhang, Xuetao Li, Qinzi E, Zuoyu Jiang, Qikun Shi, Yu Huang, Jian Wang, Yulun Huang

**Affiliations:** 1 Department of Neurosurgery, Dushu Lake Hospital Affiliated of Soochow University, Suzhou, 215000 China; 2 Department of Neurosurgery, The First Affiliated Hospital of Soochow University, Suzhou, 215000 China; 3 Department of Neurosurgery, TaiCang Hospital of Traditional Chinese Medicine, Suzhou, 215000 China

**Keywords:** Glioblastoma, KCNA1, SLC7A11, Ferroptosis, Tumor invasion

## Abstract

**Background:**

The high invasiveness and infiltrative nature of Glioblastoma (GBM) pose significant challenges for surgical removal. This study aimed to investigate the role of KCNA1 in GBM progression.

**Methods:**

CCK8, colony formation assay, scratch assay, transwell assay, and 3D tumor spheroid invasion assays were to determine how KCNA1 affects the growth and invasion of GBM cells. Subsequently, to confirm the impact of KCNA1 in ferroptosis, western blot, transmission electron microscopy and flow cytometry were conducted. To ascertain the impact of KCNA1 in vivo, patient-derived orthotopic xenograft models were established.

**Results:**

In functional assays, KCNA1 promotes the growth and invasion of GBM cells. Besides, KCNA1 can increase the expression of SLC7A11 and protect cells from ferroptosis. The vivo experiments demonstrated that knocking down KCNA1 inhibited the growth and infiltration of primary tumors in mice and extended survival time.

**Conclusion:**

Therefore, our research suggests that KCNA1 may promote tumor growth and invasion by upregulating the expression of SLC7A11 and inhibiting ferroptosis, making it a promising therapeutic target for GBM.

**Supplementary Information:**

The online version contains supplementary material available at 10.1186/s12935-023-03199-9.

## Introduction

Glioblastoma multiforme is the most common malignant primary brain tumor, and its treatment include surgical resection, radiotherapy, chemotherapy, and electric-field therapy [1, 2]. Owing to the highly infiltrative nature of GBM cells and their potential origin in specific functional areas of the brain, complete surgical resection is often impossible [3]. Therefore, glioblastoma still lacks an effective treatment and is characterized by high recurrence rates, high mortality, and poor prognosis [4]. Investigating the molecular mechanisms and biological behavior underlying glioblastoma cell invasion and metastasis is important in the search for novel therapeutic targets [5]. We found KCNA1 is overexpressed in GBMi^inv^ cells in vivo in patient-derived orthotopic xenograft (PDOX) models and in patient glioblastoma tumor, functioning as a downstream target of microRNAs in GBM cells to promote tumor invasion and metastasis [6]. Besides, KCNA1 role in cancer reportedly includes potential regulation of mitochondrial function in HeLa cells [7] and indicator of poor prognosis in colorectal cancer [8].

The KCNA1 gene belongs to a large family of genes that provide instructions for making potassium channels. These channels, which transport positively charged potassium atoms (potassium ions) into and out of cells, play a key role in a cell's ability to generate and transmit electrical signals. Potassium ion channels are widely expressed ion channels on cell and organelle membranes and are essential for maintaining central nervous system function [9]. Among them, as a voltage-gated potassium channel, Kv1.1 plays an important role in controlling neuronal excitability [10, 11]. More and more research suggest that potassium ion channels regulate the growth of primary tumors, epithelial-mesenchymal transition (EMT), angiogenesis, and invasion [12–15].

Ferroptosis is a regulatory cell death process that is regulated through specific signal transduction pathways [16]. With extended lipid peroxidation arising from reactive oxygen species (ROS), many studies have indicated that ferroptosis has different effects on tumor progression and response [17, 18]. Therefore, targeting ferroptosis could be a potential strategy for treating cancers [19, 20]. SLC7A11 is an important regulatory protein in the ferroptosis pathway that increases glutathione (GSH) biosynthesis to detoxify products of lipid peroxidation [21]. SLC7A11 is regulated by various signaling pathways, such as P53, activating transcription factor-4, signal transducers, activators of transcription, and nuclear factor erythroid 2-related factor [22–25]. Therefore, this study aimed to propose a new mechanism by which KCNA1 regulates the expression of SLC7A11 in glioblastoma.

In this study, we confirmed that KCNA1 expression was associated with proliferation and invasion of GBM cells. Meanwhile, our study demonstrated firstly that decreased KCNA1 expression promotes ferroptosis through SLC7A11-GPX4 pathway inhibition. Our study has shown that KCNA1 rescues GBM cells from ferroptosis and promoting tumor progression. Therefore, KCNA1 could be a promising medical target for GBM treatment.

## Materials and methods

### Cell lines and cell culture

Primary cell lines, SHG140, CXM, and LFL, derived from GBM samples, were obtained from the First Affiliated Hospital of Soochow University, cultured, and identified using short tandem repeat DNA profiling. U87, U251 and T98 cell lines were obtained from the Shanghai Academy of Biological Sciences. All the cells were cultured in the DMEM medium supplemented with 10% fetal bovine serum (FBS).

### Bioinformatic analysis

We analyzed KCNA1 expression in different aera of glioma using the database of the Ivy Glioblastoma Atlas Project [26]. The raw data was used for bioinformatic information analysis.

### Cell counting kit-8 (CCK-8) and colony formation assay

For the CCK-8 assay, cells with good growth condition (3000 cells/well) were placed in 96-well plates with 100 μL of culture medium. After adding approximately 10 μL of CCK-8 reagent (CA1210, Solarbio) to each well and incubating for 3 h at 37 °C, absorbance at wave-length 450 nm was measured and recorded for further analysis. For the colony formation assay, cells (1000 cells/well) were placed in 6-well plates with 2 mL of culture medium. Then the cells were cultured for 14 days, with the solution changed every 3 days. After fixing the colonies with 4% paraformaldehyde for 15 min, stain with 0.5% crystal violet for 20 min.

### Short hair pain RNA (shRNA) and small interfering RNA (siRNA) transfections

KCNA1-shRNA knockdown and lentivirus overexpression (OE) of KCNA1 were designed and constructed by Genechem (China), transfected into GBM cells, and selected using puromycin to obtain stable transfected cells. After 5 days, we achieved stable knockdown or overexpression of the KCNA1 gene in glioblastoma cells. Then, cells were further cultured to obtain a sufficient quantity for subsequent experiments. SLC7A11-siRNAs (siSLC7A11) and negative control were designed and chemically synthesized by GenePharma (China). Western blotting was performed to determine transfection efficiency. The shRNAs and siRNAs sequences used for transfection are listed in Additional file 5: Table S1.

### Apoptosis analysis

Apoptosis was detected using an annexin V-FITC/PI staining kit (BD Biosciences, NJ, USA). After digestion and centrifugation, the cells were resuspended in 100 μL of binding buffer. Subsequently, Annexin V-FITC (5 μL) and PI (5 μL) were added. The mixture was then incubated in the dark for 15 min, followed by detection using a flow cytometer and raw data analysis using FlowJo V10.81.

### Western blotting

We used radio immunoprecipitation assay (RIPA) lysis buffer (Beyotime, China) to extract total protein from cells. After centrifuging with 12000 g for 15 min at 4 ℃, the supernatant was to determine the protein concentration using a bicinchoninic acid (BCA) assay kit (Beyotime, China). Subsequently, proteins were electrophoresed to achieve complete separation and then transferred onto a polyvinylidene fluoride (PVDF) membrane at specific time intervals according to their molecular weights. The PVDF membrane was subsequently sealed with 5% bovine serum albumin (BSA) at 23 °C for 1 h, washed thoroughly using phosphate buffer saline with tween-20 (PBST) thrice, and incubated with primary antibody at 4 °C overnight. Following this, it was washed three times with PBST and then incubated with a peroxidase-conjugated secondary antibody at 23 °C for 1 h on the second day. Then, chemiluminescent (ECL) reagent was added to make them visualize. ImageJ software was employed to calculate the grayscale value.

### Immunofluorescence

Cells were seeded onto slides, fixed with 4% paraformaldehyde for 20 min at an appropriate density. After washing with PBST three times, permeabilize with 0.3% Triton X-100 for 20 min. Then, the cells were blocked at 23 °C with 5% BSA for 1 h. Thereafter, the cells were incubated with primary antibodies overnight at 4 °C and exposed to secondary antibodies for 1 h at 23 °C. Finally, transfer the cover glass onto the slide with DAPI (Southernbiotech, China). Images were captured using a confocal fluorescence microscope (Leica).

### Wound healing and transwell assays

SHG140 and U87 cells were inoculated into a 6-well plate to evaluate the migration capability. Horizontal lines were drawn using a 100-µL pipette tip, and the plate was replaced with a serum-free culture medium when the cells filled up the plate. After 0 h, 24 h and 48 h, capture Images and calculate migration distance. For cell invasion assays, 30000 cells were evenly spread into the upper chamber containing Matrigel (Corning, USA), whereas the lower chamber were full of medium containing 10% FBS to induce tumor cell invasion. After invasion for 48 h, the invading tumor cells were fixed with 4% paraformaldehyde for 20 min, subsequently stained with crystal violet for 20 min. After natural air-drying, capture pictures and count the number of cells using ImageJ.

### Spheroid and organoid assays

5000 cells were placed in a 96-well ultra-low attachment culture dish (Corning Cat. No. 4515) and then centrifuged at 200 g for 5 min to allow the cells to gather together. Spheroids could be harvested after 3 days. At the same time, organoids were cultivated as previously described [27]. For the invasion assay, the culture medium in the plate was aspirated and replaced with a culture medium containing Matrigel (Corning, USA) for 2 days. Images were captured using an optical microscope, and the invasion distances of the leading-edge cells and number of edge antennae were evaluated.

### RNA-sequencing and bioinformatics analysis

Extract total RNA using Trizol reagent and evaluate and check the quality of RNA. After total RNA extraction, eukaryotic cell mRNA was enriched using Oligo(dT) beads. Subsequently, the enriched mRNA was fragmented into short fragments and subjected to reverse transcription to generate cDNA. Then cDNA fragment ends were ligated with Illumina sequencing adapters. The size of the linked fragments was selected by agarose gel electrophoresis and polymerase chain reaction (PCR) amplification. The cDNA library was subjected to sequencing using an Illumina Novaseq6000 sequencer provided by Gene Denovo Biotechnology Co. (Guangzhou, China). Differential expression and pathway analysis was performed using gene set enrichment analysis (GSEA). Besides, we downloaded the expression data from publicly available databases, the TCGA and the CGGA and the correlation between genes was calculated using the R package.

### Determination of JC-1, ROS level, and lipid peroxidation

Cells in good condition were digested and resuspended to 5 × 10^4^ cells in flow cytometry tubes per tube. The cells were then incubated with DCFH-DA (Beyotime, China), JC-1 (Beyotime, China), C11-BODIPY 581/591 (Abclonal, China) and MitoSOX (M36008, Thermo Fisher) in a serum-free medium for suitable time according to the manufacturer’s instructions. Subsequently, cells were washed thrice with PBS and detected using flow cytometry. Finally, raw data were analyzed using FlowJo V10.81.

### Transmission electron microscopy (TEM)

The cells were digested, centrifuged at 500 g for 5 min, and fixed with 2.5% glutaraldehyde solution at 4 °C overnight. Thereafter, they were fixed with osmium acid and uranium acetate and were dehydrated using different concentrations of alcohol and acetone. The samples were then embedded in a resin mixture. Ultra-thin sections with 60–80 nm thickness were cut, stained using a uranium-lead double staining method, and observed at 120 kV using TEM (Tecnai, USA). Images were acquired for further analysis.

### Determination of ATP and GSH/oxidized glutathione (GSSH) assay

Adenosine triphosphate (ATP) and GSH levels in SHG140 and U87 cells were measured using enhanced ATP assay kit (Beyotime, S0027, China) and GSH/GSSG assay kit (Beyotime, S0053, China). Each experiment had been repeated three times.

### Animal experiment

BALB/c nude mice (4–5 weeks old, male) in this study were purchased from the Institute of Oncology, Chinese Academy of Medical Sciences (Beijing, China) and raised in a pathogen-free environment. A total 1 × 10^6^ U87 cells with luciferase-encoded lentivirus (Gene Chem, China) were injected the frontal subdural region of mice. Intracranial tumor growth was recorded using The IVIS Imaging System (Caliper Life Sciences) after injecting with D-luciferin (Solarbio, China) on days 7, 14 and 28. Once the mice died during the experiment, we took out their brains and fixed them using 4% paraformaldehyde. In the end, all the samples would be embedded and sliced for hematoxylin and eosin (H&E) staining and immunohistochemical assay (IHC). Guidelines of the Ethics Committee of Soochow University were complied with during all animal studies.

### Statistical analysis

All experimental data acquired were repeated at least three times and values were presented as mean and standard deviation (mean ± SD). GraphPad Prism 9 software was used for statistically analysis. To analyze the differences between groups, we used log-rank test, t-test and one-way ANOVA test. P < 0.05 is considered statistically significant.

## Results

### KCNA1 is overexpressed in leading edge of GBM

**Fig. 1:**
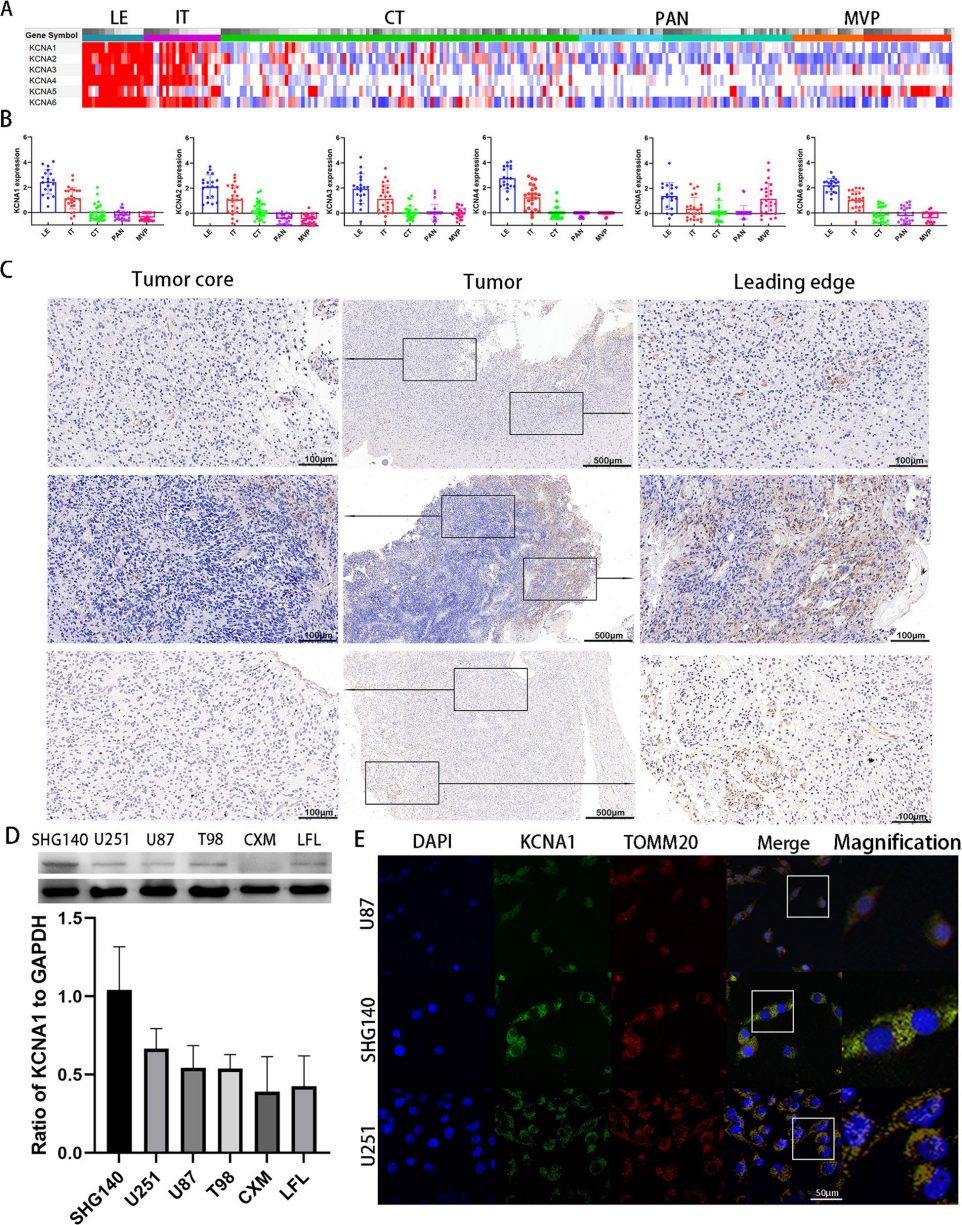
KCNA1 is upregulated at the Leading Edge **A**–**B** the heatmap (**A**) and histogram (**B**) of KCNA1–KCNA6 mRNA expression in the Ivy Glioblastoma Atlas Project (**C**) immunohistochemical staining of GBM tissue in the tumor core and leading edge (the middle and rest scale bar are 500 μm and 100 μm, respectively). **D** Western blot to detect KCNA1 expression in different cell lines. Data are expressed as standard deviation. **E** Immunofluorescence assay of KCNA1 and TOMM20 in different cell lines (the scale bar is 50 μm)

According to the Ivy Glioblastoma Atlas Project, 122 RNA samples were divided into five structures: leading edge (LE), infiltrating tumor (IT), cellular tumor (CT), microvascular proliferation (MVP), and pseudo palisading cells around necrosis (PAN) as identified by H&E staining. We observed that leading edge and infiltrating tumors had higher expression of the KCNA family, including KCNA1–6 (Fig. 1A, B), which is consistent with the conclusion of a previous study that KCNA1 is highly expressed in marginally invasive cells [6]. Simultaneously, we collected GBM samples from the First Affiliated Hospital of Soochow University and confirmed the high expression of KCNA1 protein at the leading edge through a comparison of IHC and H&E staining (Fig. 1C and Additional file 1: Fig. S1A). Subsequently, three patient-derived primary cell lines, SHG140, CXM, and LFL, and common GBM cell lines, U251, U87, and T98, were used for western blotting (Fig. 1D). Finally, we selected SHG140 and U87 cells for further experiments. Moreover, immunofluorescence staining revealed that KCNA1 was associated with the mitochondrial marker antibody, TOMM20, regarding cellular spatial positioning (Fig. 1E). Summarily, KCNA1 is highly expressed at the tumor leading edge and may be related to the mitochondria in GBM cells.

### KCNA1 knockdown inhibits GBM proliferation and invasion

**Fig. 2:**
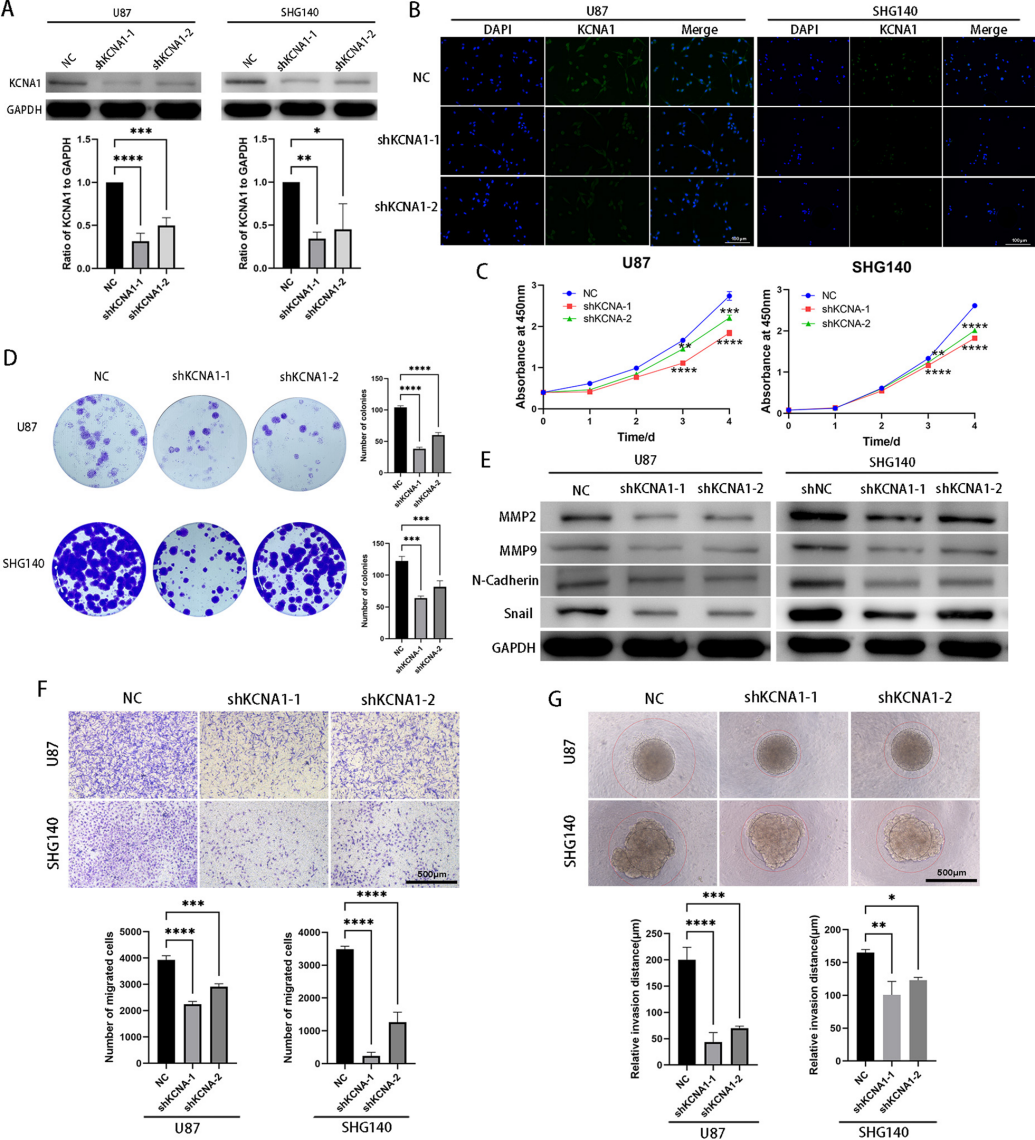
KCNA1 knockdown inhibits GBM proliferation and invasion. **A** Western blot to verify KCNA1 knockdown efficiency, **B** Immunofluorescence to verify KCNA1 knockdown efficiency (the scale is bar 100 μm). **C** CCK-8 assay for cell viability of SHG140 and U87 cells after transfection (n = 3), **D** Colony formation assays of SHG140 and U87 cells to measure the proliferation capacity (n = 3). **E** Western blot to detect expression of MMP2, MMP9, N-Cadherin and Snail in SHG140 and U87 after transfection, **F** Transwell assay of SHG140 and U87 cells after transfection (Scale bar = 500 μm), **G** 3D tumor spheroid invasion assay to assess invasion of SHG140 and U87 cells (Scale bar = 500 μm). One-way ANOVA for multi-group comparisons. n = 3, *p < 0.05, **p < 0.01, ***p < 0.001, ****p < 0.0001. GBM, glioblastoma; shKCNA1, KCNA1- shorthair pin RNA

The Ivy Glioblastoma Atlas Project suggests that high KCNA1 expression may be associated with glioblastoma progression. To determine the potential role of KCNA1 in GBM development, we knocked down KCNA1 in SHG140 and U87 cell lines with two shRNA (shKCNA1-1 and shKCNA1-2). Western blotting and immunofluorescence assay confirmed that the knockdown efficiency in both cell lines (Fig. 2A, B). Compared to the control group, low expression of KCNA1 significantly reduced cell proliferation, and the number of formed clones decreased simultaneously (Fig. 2C, D). Increased apoptosis was observed after knockdown of KCNA1 (Additional file 2: Fig. S2A). Moreover, proteins involved in EMT, including MMP2, MMP9, N-cadherin, and Snail, were reduced in the shKCNA1 cells (Fig. 2E). The migration and invasion capacity of KCNA1 knockdown cells were reduced (Fig. 2F and Additional file 2: Fig. S2B). In addition, the three-dimensional (3D) tumor model mimics the complex microenvironment and architecture of tumors in vitro better than the traditional two-dimensional (2D) cell culture models. Therefore, we examined the invasion of U87 and SHG140 spheroids and confirmed that more KCNA1 proteins were associated with a stronger invasive ability, which was consistent with the results of the organoid invasion assays (Fig. 2G and Additional file 2: Fig. S2C).

### KCNA1 promotes GBM progression in vitro

**Fig. 3:**
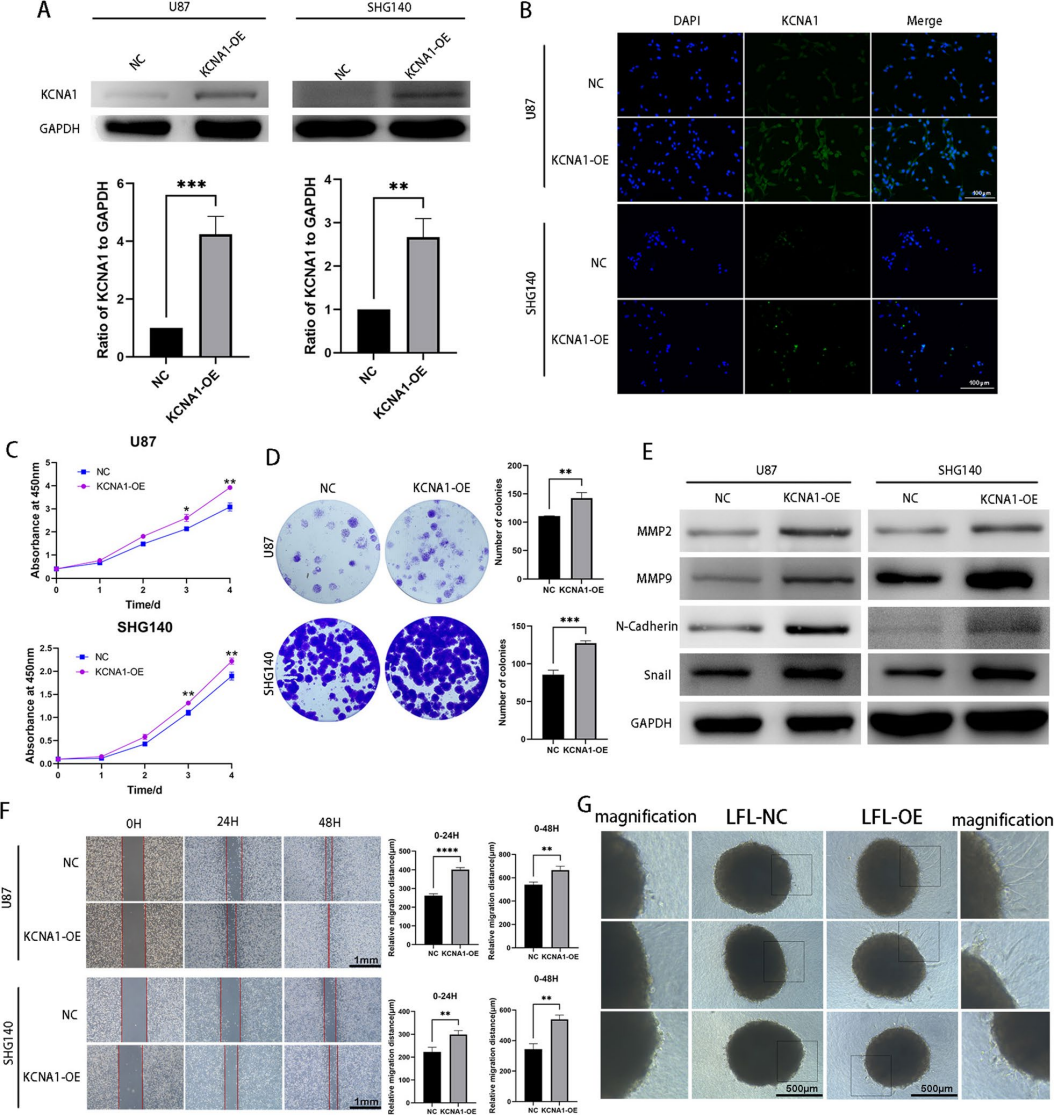
KCNA1 promotes GBM progression in vitro. **A** Western blot to verify KCNA1 overexpress efficiency, **B** Immunofluorescence to verify KCNA1 overexpress efficiency (the scale is bar 100 μm). **C** CCK-8 assay for cell viability of SHG140 and U87 cells after transfection (n = 3), **D** Colony formation assays of SHG140 and U87 cells to measure the proliferation capacity (n = 3). **E** Western blot to detect expression of MMP2, MMP9, N-Cadherin and Snail in SHG140 and U87 cells after transfection, **F** scratch assay of SHG140 and U87 cells after transfection (Scale bar = 1 mm), **G** Organoids assay transfected with NC and KCNA1-OE (Scale bar = 500 μm). Student’s t-test for two-group comparison. n = 3, **p < 0.01, ***p < 0.001, ****p < 0.0001

We overexpressed the KCNA1 gene in SHG140 and U87 cells, and substantiated the overexpression efficiency through western blotting and immunofluorescence assay (Fig. 3A, B). Using CCK-8 assays and colony formation experiments, we demonstrated that KCNA1 enhanced the proliferative ability of GBM cells (Fig. 3C, D). The proportion of apoptotic cells decreased upon overexpression of KCNA1 (Additional file 3: Fig. S3A). Western blot analysis was used to validate the increased expression of proteins associated with tumor invasion in KCNA1-OE cells (Fig. 3E). Furthermore, we validated in both 2D and 3D models that KCNA1-OE cells exhibit increased invasion and migration abilities (Fig. 3F, G, and Additional file 3: Fig. S3B, C).

### SLC7A11 is downregulated after KCNA1 knockdown in GBM cells

**Fig. 4:**
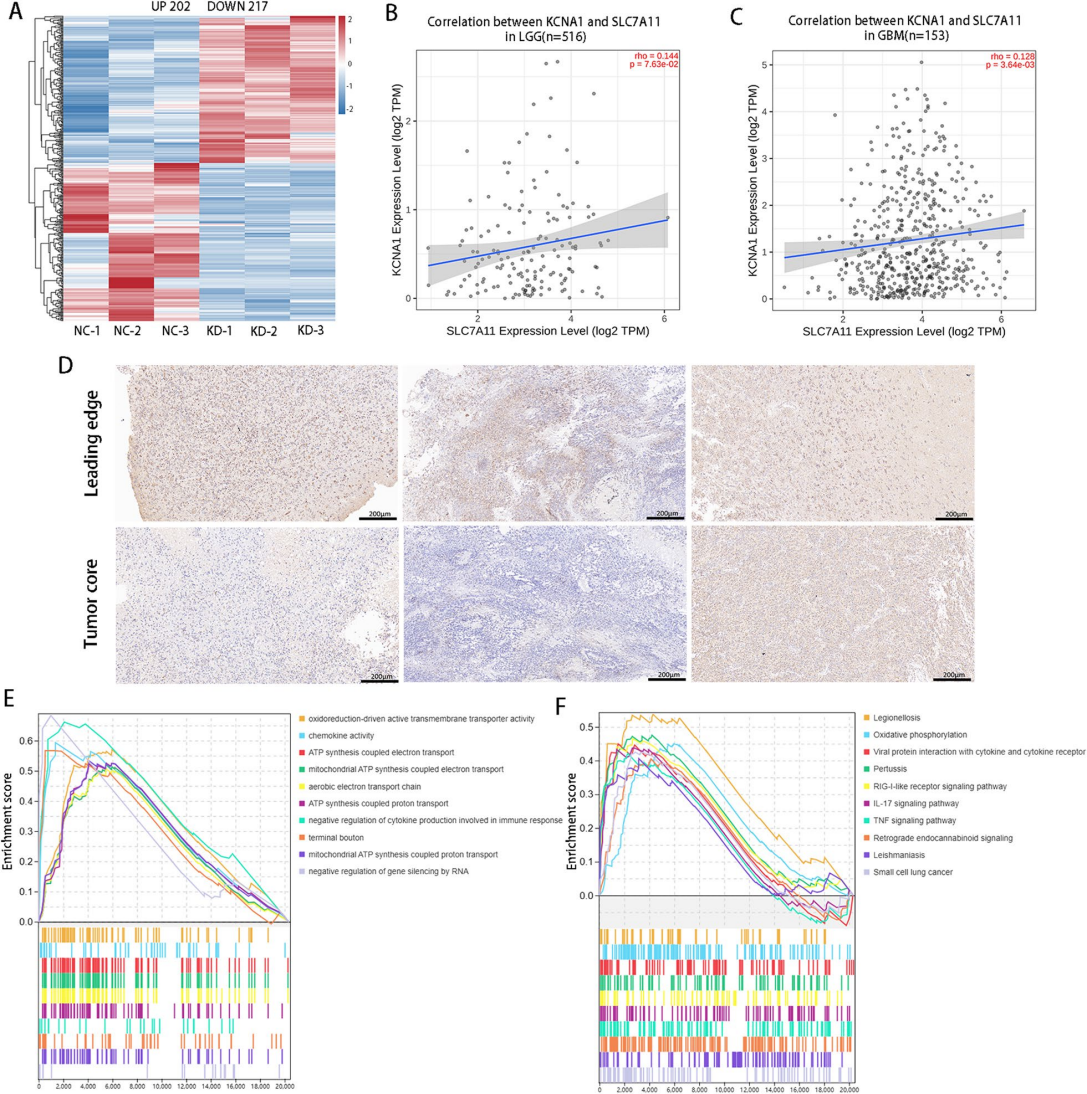
SLC7A11 is downregulated after KCNA1 knockdown and the GSEA of RNA sequencing. **A** Heatmap of differentially expressed genes of NC and sh-KCNA1. **B** Correlation analysis of KCNA1 and SLC7A11 genes in the TCGA database for low-grade glioma (LGG), **C** Correlation analysis of KCNA1 and SLC7A11 genes in the TCGA database for GBM, **D** IHC staining of SLC7A11 in the leading edge and tumor core, **E** Gene Ontology enrichment analysis, **F** KEGG pathway enrichment analysis

To further determine the specific mechanisms by which KCNA1 influences glioblastoma progression, we conducted RNA sequencing in the NC and sh-KCNA1 groups. The results revealed 202 upregulated genes and 217 downregulated genes between the two groups (Fig. 4A). The top 20 genes were selected based on the criteria of P < 0.05 and |log2FC|> 2 (Additional file 6: Table S2). Among these, the SLC7A11 was identified, with substantial literature evidence, to be involved in ferroptosis and tumor progression. The correlation between KCNA1 and SLC7A11 was positive, as investigated using the Cancer Genome Atlas Program (TCGA) database (Fig. 4B, C). Furthermore, we confirmed that SLC7A11 expression was higher at the leading edge than in the tumor core in the collected tumor samples (Fig. 4D), which is consistent with our previous findings that KCNA1 distribution was higher at the tumor edge, suggesting that KCNA1 may positively regulate the expression of SLC7A11. Finally, to better understand the role of KCNA1 in tumors, we conducted a Gene Set Enrichment Analysis (GSEA). Gene ontology (GO) enrichment analysis revealed that KCNA1 might be associated with mitochondrial-related functions, including oxidoreduction-driven active transmembrane transporter activity, ATP synthesis-coupled electron transport, mitochondrial ATP synthesis-coupled electron transport, ATP synthesis-coupled proton transport, and mitochondrial ATP synthesis-coupled proton transport (Fig. 4E). Kyoto Encyclopedia of Genes and Genomes (KEGG) enrichment analysis indicated that KCNA1 was associated with oxidative phosphorylation and interleukin-17 (IL-17) and tissue necrosis factor (TNF) signaling pathways (Fig. 4F).

### KCNA1 inhibits ferroptosis in GBM cells

**Fig. 5:**
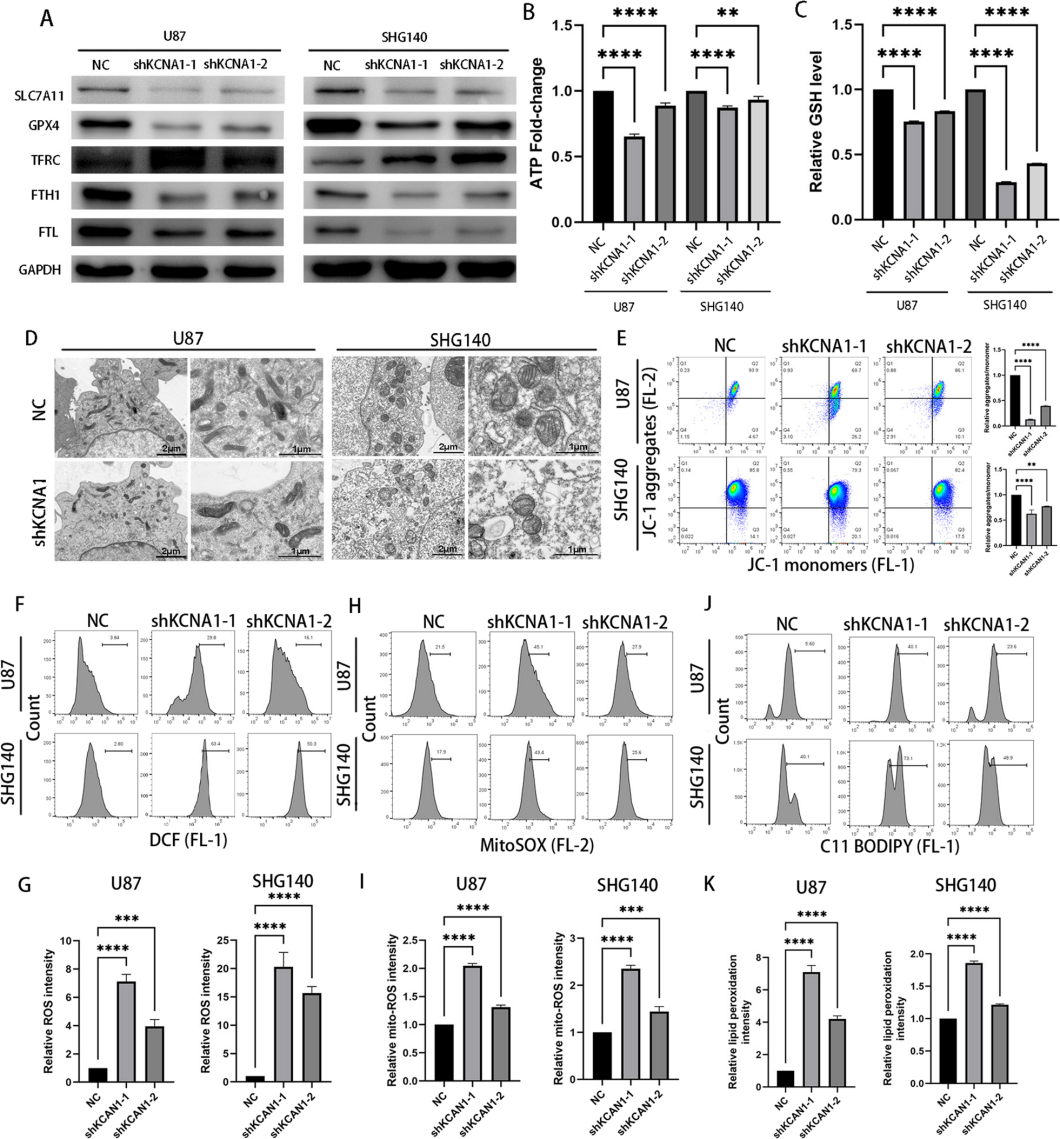
Knockdown of KCNA1 promotes ferroptosis. **A** Expression of ferroptosis-associated proteins cells after transfection, **B** ATP produced by U87 and SHG140 cells was detected using an enhanced ATP assay kit, **C** The GSH in U87 and SHG140 cells was detected using a GSSG/GSH assay kit, **D** Transmission electron microscopy in U87 and SHG140 cells. **E** Mitochondrial membrane potential was detected using a JC-1 assay kit, **F**–**G** Intracellular ROS level was detected using a DCFH-DA assay kit, **H**–**I** Intramitochondrial ROS level was detected using a MitoSOX assay kit, **J**–**K** The accumulation of lipid ROS was detected using a C11-BODIPY 581/591 assay kit. One-way ANOVA for multi-group comparisons. n = 3, **p < 0.01, ***p < 0.001, ****p < 0.0001

To confirm further the relationship between SLC7A11 and KCNA1 in U87 and SHG140 cell lines, we conducted a western blot analysis and confirmed that KCNA1 knockdown decreased SLC7A11 expression. SLC7A11 regulates the entry of cystine into the cells, and subsequently promoting GSH production, which plays a role in combating ferroptosis. Within this pathway, GPX4 is the enzyme that regulates GSH generation, and our experiments also verified reduced GPX4 expression in KCNA1 knockdown cells. We found that iron-related transport proteins, including the transferrin receptor (TFRC) responsible for transporting Fe^3+^ into cells, were increased in knockdown cells. In addition, iron ions are stored in the cells as heavy and light chains ferritins (FTH1 and FTL). The two proteins, known for their protective role against ferroptosis[28], found to have low expression in KCNA1 knockdown cells (Fig. 5A). Furthermore, after KCNA1 knockdown, ATP production and GSH levels decreased (Fig. 5B, C). To investigate the putative role of KCNA1 in the ferroptosis, we used transmission electron microscopy (TEM) to observe morphological changes in mitochondrion following KCNA1 knockdown. Compared with that in the control group, a significant reduction was observed in the number of mitochondrial cristae and mitochondrial matrix exhibited vacuolization in the KCNA1 knockdown group (Fig. 5D). Additionally, using flow cytometry, we detected decreased mitochondrial membrane potential (Fig. 5E), increased intracellular ROS level (Fig. 5F, G), and heightened level of mitochondrial-targeted ROS (Fig. 5H, I), along with accumulated lipid ROS (Fig. 5J, K) after KCNA1 knockdown. These results collectively confirmed a high level of ferroptosis in KCNA1-knockdown cells.

### Knockdown of SLC7A11 inhibits tumor progression and promotes ferroptosis

**Fig. 6:**
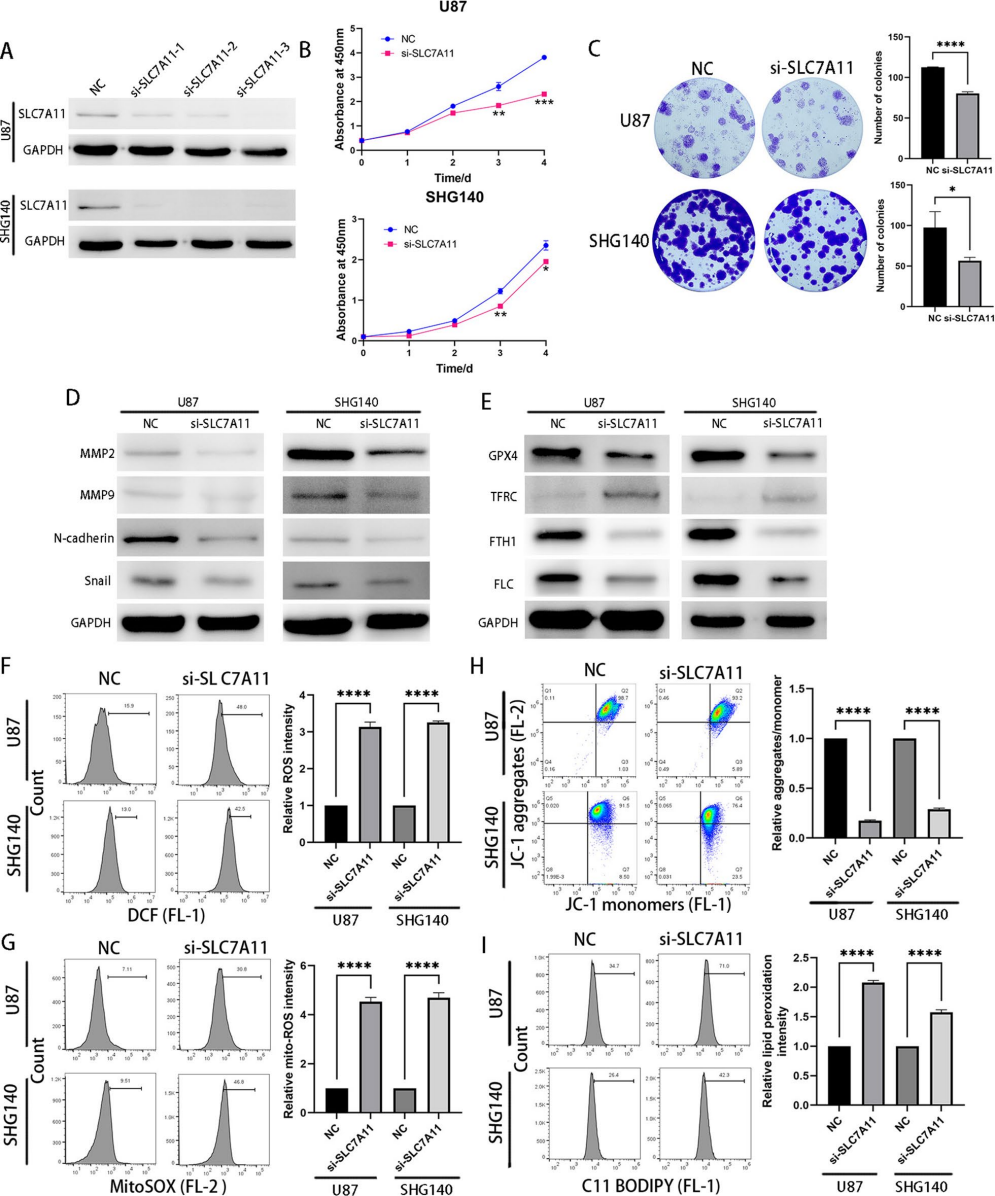
Knockdown of SLC7A11 inhibits tumor progression and promotes ferroptosis. **A** Western blot analysis for siRNA knockdown efficiency of SLC7A11, **B** CCK-8 assay for cell viability, **C** Colony formation experiments for cell viability, **D** Expression of EMT-associated proteins, **E** Expression of ferroptosis-associated proteins, **F**–**I** Measurement of intracellular ROS level (**F**) intramitochondrial ROS level (**G**) mitochondrial membrane potential (**H**) and accumulation of lipid ROS (**I**). Student’s t-test for two-group comparison. n = 3, *p < 0.05, ***p < 0.001, ****p < 0.0001

SLC7A11 downregulation coincided with downregulation of KCNA1. Therefore, we investigated the role of SLC7A11 in U87 and SHG140 cells. Firstly, three siRNAs were designed for transfection, and their knockdown efficiency was estimated using western blotting (Fig. 6A). Ultimately, si-SLC7A11-3 was selected for subsequent experiments. CCK-8 assays and colony formation experiments revealed that SLC7A11 inhibits the proliferation of GBM cells (Fig. 6B, C). Scratch and transwell assays demonstrated that SLC7A11 suppressed the migration and invasion of GBM cells (Additional file 4: Fig. S4A, B). Furthermore, western blotting confirmed the reduced expression of EMT-associated proteins in siRNA-transfected cell lines (Fig. 6D). Finally, we validated, through western blot and flow cytometry, the role of SLC7A11 in inhibiting ferroptosis in glioblastoma (Fig. 6F, I). Thus, the aforementioned results indicate that SLC7A11 promotes tumor progression and suppresses ferroptosis in GBM cells, exerting a similar role to that of KCNA1.

### KCNA1 confers a protective effect against ferroptosis

**Fig. 7:**
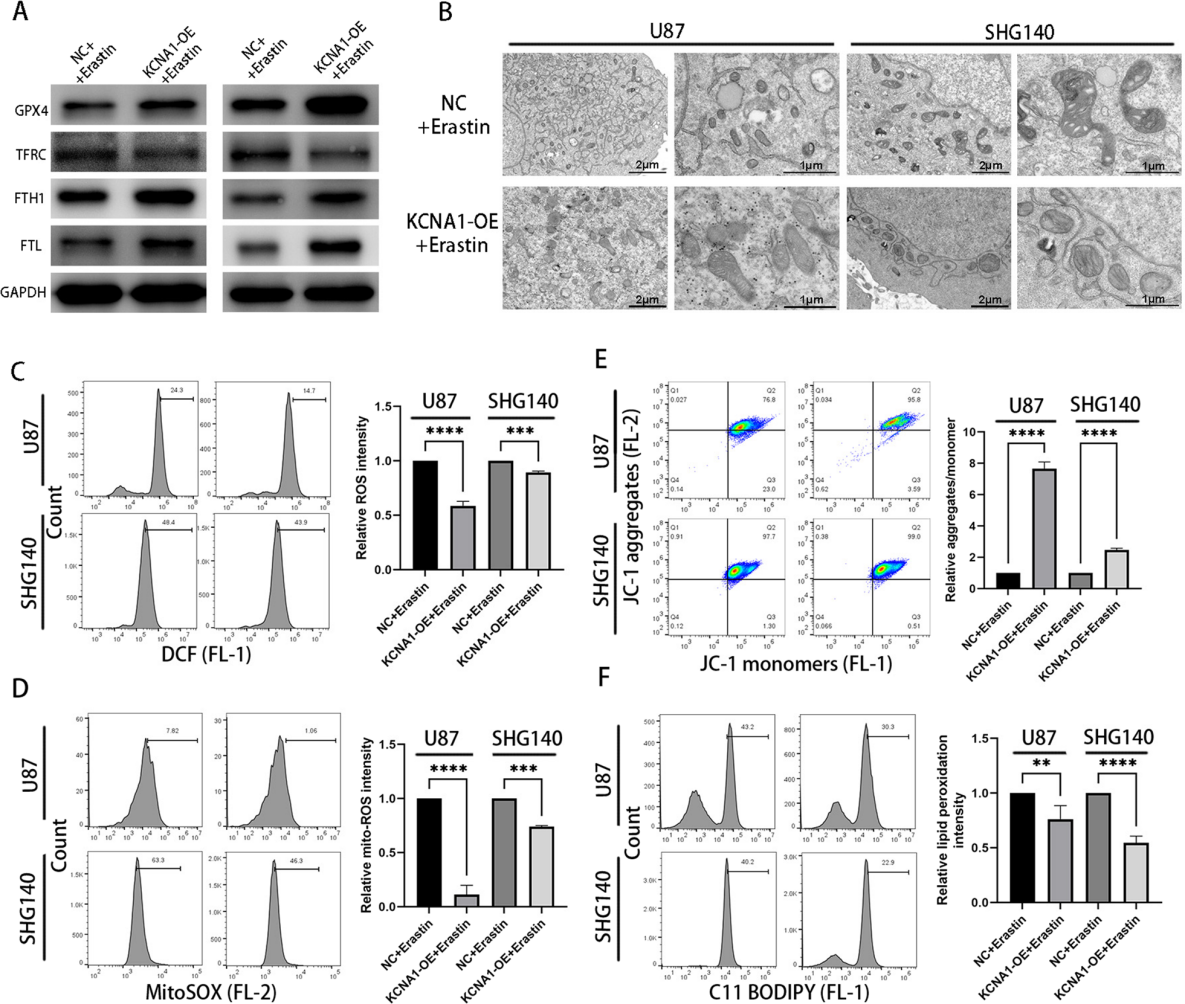
KCNA1 protects against ferroptosis. **A** Western blot analysis for detecting protein expression related to ferroptosis, **B** Transmission electron microscopy **C**–**F** Measurement of intracellular ROS level (**C**) intramitochondrial ROS level (**D**) mitochondrial membrane potential (**E**) and accumulation of lipid ROS (**F**). Student’s t-test for two-group comparison. n = 3, **p < 0.01, ***p < 0.001, ****p < 0.0001

In order to gain further insights into the role of KCNA1 in ferroptosis, we induced ferroptosis in glioblastoma cells using the drug Erastin. Compared with the control group, cells overexpressing KCNA1 showed increased expression of GPX4, FTH1, and FTL proteins, while the expression of TFRC protein was reduced (Fig. 7A). Furthermore, transmission electron microscopy confirmed a decrease in mitochondrial cristae and vacuolization in response to ferroptosis, which were reversed by KCNA1 overexpression (Fig. 7B). Additionally, GBM cells with higher KCNA1 expression exhibited increased mitochondrial membrane potential, decreased intracellular and mitochondrial ROS levels, and reduced lipid peroxidation during ferroptosis (Fig. 7C, D, E and F). These results demonstrated the protective role of KCNA1 against intracellular ferroptosis.

### KCNA1 inhibits tumor progression in vivo

**Fig. 8:**
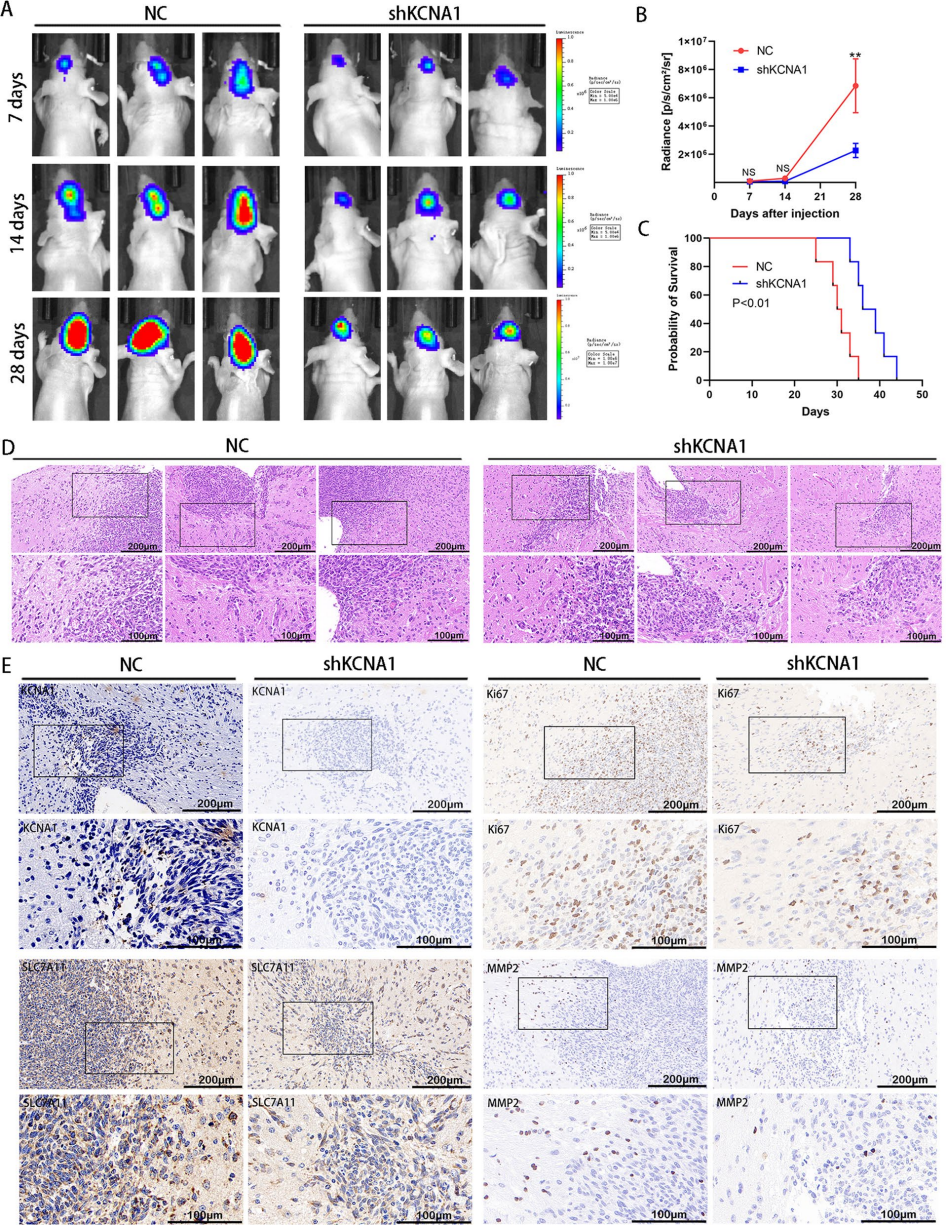
KCNA1 inhibits tumor progression in vivo. **A** Representative images of bioluminescence of mice injected with cells, **B** Radiance analysis of these mice for the NC and shKCNA1 group. Student’s t-test for two-group comparison. n = 6, **p < 0.01, **C** Survival curves of the two groups, log-rank test, **D** H&E staining of mice brain slices, the upper scare bar = 200 μm, the below scare bar = 100 μm **E** IHC staining of mouse brain sections, the upper scare bar = 200 μm, the below scare bar = 100 μm

In vivo, we implanted stable transfections of negative control virus (NC) and KCNA1-interfering lentivirus (shKCNA1) U87 cells into the mouse brains to simulate the growth of glioblastoma. Using a bioluminescence imaging system on days 7, 14, and 28 post-implantation, we observed tumor size (Fig. 8A). The tumors in the shKCNA1 group were smaller than that in the NC group (Fig. 8B), and mice in the former group exhibited longer tumor-bearing survival times (Fig. 8C). Subsequent H&E staining revealed larger intracranial tumors with rougher tumor edges in the NC group, indicating a stronger invasive behavior of tumor cells into the surrounding tissue (Fig. 8D). Immunohistochemistry (IHC) analysis of mouse brain sections showed reduced expression of KCNA1, Ki67, SLC7A11, and MMP2 proteins in the shKCNA1 group, consistent with the in vitro experimental data (Fig. 8E). These findings suggest that KCNA1 promotes glioblastoma growth and invasion in vivo, and may inhibit tumor ferroptosis through its interaction with SLC7A11.

## Discussion

In this study, we validated that KCNA1 promotes cell growth and invasion of glioblastoma cells both in vivo and in vitro. Additionally, we demonstrated for the first time that KCNA1 inhibits ferroptosis in glioblastoma by positively regulating the expression of SLC7A11. Hence, this study provides a novel approach for potential therapeutic intervention against glioblastoma by targeting the KCNA1-SLC7A11 axis to inhibit tumor progression.

In previous studies, KCNA1 was recognized for its crucial role in the central nervous system [29, 30]. The Kv1.1 channel primarily facilitates the transport of potassium ions into neuronal cells, thereby regulating membrane potential and signal transmission [31]. This crucial role helps prevent excessive excitation of neurons. For instance, upregulating Kv1.1 through CRISPRa significantly reduces neuronal excitability [32, 33]. Moreover, KCNA1 mutations can lead to abnormal Kv1.1 function, primarily associated with Episodic Ataxia Type 1 (EA1) and epilepsy [34, 35]. In vivo, mice with KCNA1 knockdown exhibit seizures, premature death, and cardiorespiratory dysfunction [36]. However, recent research on glioblastoma indicated that KCNA1 promotes the invasion of tumor cells in highly infiltrative and invasive cell population at the tumor edge [6]. Moreover, KCNA1 was upregulated during seizures and tumor formation in glioblastoma models [34]. In this study, we confirmed that KCNA1 promoted the growth, migration, and invasion of GBM cells in both two and three-dimensional environments. In contrast, considering previous research that demonstrated a significant association between KCNA1 overexpression and the onset of epilepsy [31, 37], our in vivo experiments did not observe any differences in ataxia attacks or the probability of seizures between the two groups of mice (NC and sh-KCNA1 groups). This might be attributed to the fact that the baseline KCNA1 expression in mice did not differ and that the differential expression of KCNA1 implanted within glioma cells only resulted in variations in the biological behavior of the tumors themselves. Considering the possibility of neurotoxic effects, drugs targeting KCNA1 for anticancer therapy should be administered at appropriate concentrations to exert anticancer effects while minimizing adverse neurological outcomes. Therefore, further research is needed to determine how to use chemical agents safely and effectively to inhibit glioblastoma progression.

Ferroptosis, a cell-death mechanism that relies on iron and lipid peroxidation, was been recently discovered [38]. In most tumor cells, ferroptosis is suppressed [39]. Therefore, investigating the mechanisms underlying various ferroptosis pathways and identifying targets for inducing ferroptosis in tumor cells, or combining them with other therapies, has emerged as a novel approach to slowing tumor progression in cancer treatment [20, 40–42]. This study showed that KCNA1 was co-localized with the mitochondrial marker protein, TOMM20, suggesting that KCNA1 may regulate mitochondrial function. As mitochondrial damage is a hallmark of ferroptosis [43], we subsequently ferroptosis-associated markers. These results indicated that KCNA1 knockdown reduced ATP production and induced ferroptosis in tumor cells. Additionally, SLC7A11, a regulatory factor of cystine, controls the generation of GSH and GPX4, thereby inhibiting lipid peroxidation [44–46]. Further research revealed that KCNA1 could upregulate the expression of SLC7A11 and simultaneously protected cells from ferroptosis. This reveals a new mechanism by which KCNA1 inhibits ferroptosis. However, further research is needed on the specific mechanism by which KCNA1 upregulates the expression of SLC7A11.

Furthermore, our research indicates that KCNA1 promotes tumor progression by inhibiting ferroptosis, which is consistent with the conclusion of other studies that ferroptosis is suppressed during cell growth and invasion [47–50]. However, other studies reported that EMT signaling enhances the migration and invasion abilities of tumor cells and increases their sensitivity to ferroptosis [51–53]. However, different signaling molecules in the EMT pathway may have varying effects on ferroptosis [28]. For example, E-cadherin can inhibit ferroptosis, whereas TWIST1 or ZEB1 may promote ferroptosis [54, 55]. Therefore, KCNA1 may increase certain EMT signaling molecules, enhancing cell migration and invasion abilities and protecting against ferroptosis. However, the specific mechanisms underlying these processes require further investigation.

Finally, we validated in vivo that the inhibition of KCNA1 in cells led to tumor suppression during intracranial growth, extended survival time in mice, and reduced the expression of SLC7A11. Therefore, our experiments elucidated the role of KCNA1 in inhibiting ferroptosis and consequently promoting tumor growth. Similarly, previous reports have shown a significant reduction in tumor invasion and metastasis in mouse models of glioblastoma following treatment with potassium channel inhibitors [6]. Therefore, this study reveals a mechanism that promotes the GBM progression and offers a promising new direction for the GBM treatment.

## Conclusion

**Fig. 9:**
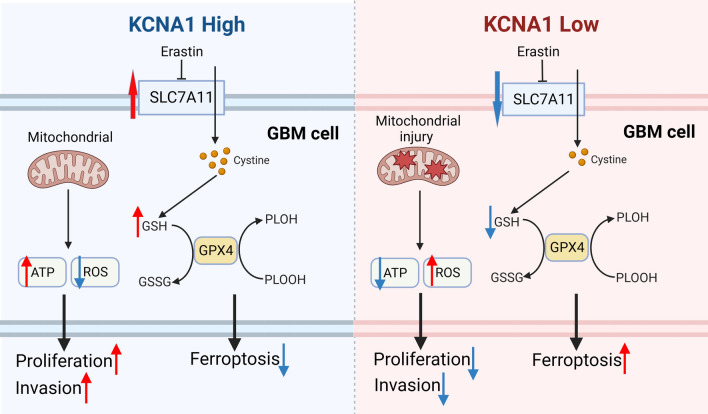
Mechanism pathway of how KCNA1 upregulates SLC7A11 to promote tumor progression

In conclusion, our experiments showed that KCNA1 promotes progression of GBM. KCNA1 knockdown can lower mitochondrial membrane potential, increase ROS level, and enhance lipid peroxidation, increasing ferroptosis. In addition, we found that KCNA1 upregulated SLC7A11. When combined with SLC7A11, it can inhibit ferroptosis, along with KCNA1's protective effect on cells undergoing ferroptosis. This study proposes that the KCNA1-SLC7A11 axis suppresses ferroptosis in glioblastoma cells, thereby promoting tumor progression (Fig. 9).

### Supplementary Information

**Additional file 1: Figure S1.** H&E staining of GBM tissue. **A** H&E staining of GBM tissue (the middle and rest scale bar are 500 μm and 100 μm, respectively).

**Additional file 2: Figure S2.** Knockdown of KCNA1 promotes apoptosis and inhibits invasion in GBM cell lines. **A** Apoptosis analysis of SHG140 and U87 cells. **B** scratch assay of SHG140 and U87 cells (Scale bar = 1 mm) **C** Organoids assay after transfection (Scale bar = 500 μm). One-way ANOVA for multi-group comparisons. n = 3, *p < 0.05, ***p < 0.001, ****p < 0.0 001.

**Additional file 3: Figure S3.** Overexpression of KCNA1 inhibits apoptosis and promotes invasion in GBM cell lines. **A** Apoptosis analysis of SHG140 and U87 cells. **B** Transwell assay of SHG140 and U87 cells after transfection (Scale bar = 500 μm), **C** 3D tumor spheroid invasion assay to assess invasion of SHG140 and U87 cells after transfection (Scale bar = 500 μm). Student’s t-test for two-group comparison. n = 3, *p < 0.05, **p < 0.01, ***p < 0.001.

**Additional file 4: Figure S4.** Knockdown of SLC7A11 inhibits the migration and invasion of GBM cells. **A** Scratch assays of U87 and SHG140 cells after transfection (Scale bar = 1 mm), **B** Transwell assays of U87 and SHG140 cells after transfection (Scale bar = 500 μm). Student’s t-test for two-group comparison. n = 3, *p < 0.05, **p < 0.01, ***p < 0.001.

**Additional file 5: Table S1.** Sequences of shRNAs and siRNAs in this study.

**Additional file 6: Table S2.** The top 20 genes.

## Data Availability

The Ivy Glioblastoma Atlas Project Dataset is available online (http://glioblastoma.alleninstitute.org/).

## References

[1] Lapointe S, Perry A, Butowski NA (2018). Primary brain tumours in adults. Lancet.

[2] Shergalis A, Bankhead A, Luesakul U, Muangsin N, Neamati N (2018). Current challenges and opportunities in treating glioblastoma. Pharmacol Rev.

[3] Karschnia P, Young JS, Dono A, Häni L, Sciortino T, Bruno F, Juenger ST, Teske N, Morshed RA, Haddad AF (2023). Prognostic validation of a new classification system for extent of resection in glioblastoma: a report of the RANO resect group. Neuro Oncol.

[4] Schaff LR, Mellinghoff IK (2023). Glioblastoma and other primary brain malignancies in adults: a review. JAMA.

[5] Le Rhun E, Preusser M, Roth P, Reardon DA, van den Bent M, Wen P, Reifenberger G, Weller M (2019). Molecular targeted therapy of glioblastoma. Cancer Treat Rev.

[6] Huang Y, Qi L, Kogiso M, Du Y, Braun FK, Zhang H, Huang LF, Xiao S, Teo WY, Lindsay H (2021). Spatial Dissection of Invasive Front from Tumor Mass Enables Discovery of Novel microRNA Drivers of Glioblastoma Invasion. Adv Sci.

[7] Liu L, Chen Y, Zhang Q, Li C (2019). Silencing of KCNA1 suppresses the cervical cancer development via mitochondria damage. Channels.

[8] Uhan S, Zidar N, Tomažič A, Hauptman N (2020). Hypermethylated promoters of genes UNC5D and KCNA1 as potential novel diagnostic biomarkers in colorectal cancer. Epigenomics.

[9] Jomova K, Makova M, Alomar SY, Alwasel SH, Nepovimova E, Kuca K, Rhodes CJ, Valko M (2022). Essential metals in health and disease. Chem Biol Interact.

[10] Feria Pliego JA, Pedroarena CM (2020). Kv1 potassium channels control action potential firing of putative GABAergic deep cerebellar nuclear neurons. Sci Rep.

[11] Ovsepian SV, LeBerre M, Steuber V, O'Leary VB, Leibold C, Oliver DJ (2016). Distinctive role of KV1.1 subunit in the biology and functions of low threshold K(+) channels with implications for neurological disease. Pharmacol Ther.

[12] Pardo LA, Stühmer W (2014). The roles of K(+) channels in cancer. Nat Rev Cancer.

[13] Hausmann D, Hoffmann DC, Venkataramani V, Jung E, Horschitz S, Tetzlaff SK, Jabali A, Hai L, Kessler T, Azoŕin DD (2023). Autonomous rhythmic activity in glioma networks drives brain tumour growth. Nature.

[14] Masi A, Becchetti A, Restano-Cassulini R, Polvani S, Hofmann G, Buccoliero AM, Paglierani M, Pollo B, Taddei GL, Gallina P (2005). hERG1 channels are overexpressed in glioblastoma multiforme and modulate VEGF secretion in glioblastoma cell lines. Br J Cancer.

[15] Catacuzzeno L, Franciolini F (2018). Role of KCa3.1 channels in modulating Ca2+ oscillations during glioblastoma cell migration and invasion. Int J Mol Sci.

[16] Stockwell BR (2022). Ferroptosis turns 10: emerging mechanisms, physiological functions, and therapeutic applications. Cell.

[17] Stockwell BR, Friedmann Angeli JP, Bayir H, Bush AI, Conrad M, Dixon SJ, Fulda S, Gascón S, Hatzios SK, Kagan VE (2017). Ferroptosis: a regulated cell death nexus linking metabolism, redox biology, and disease. Cell.

[18] Mou Y, Wang J, Wu J, He D, Zhang C, Duan C, Li B (2019). Ferroptosis, a new form of cell death: opportunities and challenges in cancer. J Hematol Oncol.

[19] Lei G, Zhuang L, Gan B (2022). Targeting ferroptosis as a vulnerability in cancer. Nat Rev Cancer.

[20] Zhang C, Liu X, Jin S, Chen Y, Guo R (2022). Ferroptosis in cancer therapy: a novel approach to reversing drug resistance. Mol Cancer.

[21] Koppula P, Zhuang L, Gan B (2021). Cystine transporter SLC7A11/xCT in cancer: ferroptosis, nutrient dependency, and cancer therapy. Protein Cell.

[22] Dodson M, Castro-Portuguez R, Zhang DD (2019). NRF2 plays a critical role in mitigating lipid peroxidation and ferroptosis. Redox Biol.

[23] Kang R, Kroemer G, Tang D (2019). The tumor suppressor protein p53 and the ferroptosis network. Free Radic Biol Med.

[24] He F, Zhang P, Liu J, Wang R, Kaufman RJ, Yaden BC, Karin M (2023). ATF4 suppresses hepatocarcinogenesis by inducing SLC7A11 (xCT) to block stress-related ferroptosis. J Hepatol.

[25] Li F, Hao S, Gao J, Jiang P (2023). EGCG alleviates obesity-exacerbated lung cancer progression by STAT1/SLC7A11 pathway and gut microbiota. J Nutr Biochem.

[26] Puchalski RB, Shah N, Miller J, Dalley R, Nomura SR, Yoon J-G, Smith KA, Lankerovich M, Bertagnolli D, Bickley K (2018). An anatomic transcriptional atlas of human glioblastoma. Science.

[27] Jacob F, Salinas RD, Zhang DY, Nguyen PTT, Schnoll JG, Wong SZH, Thokala R, Sheikh S, Saxena D, Prokop S (2020). A patient-derived glioblastoma organoid model and biobank recapitulates inter- and intra-tumoral heterogeneity. Cell.

[28] Chen X, Kang R, Kroemer G, Tang D (2021). Broadening horizons: the role of ferroptosis in cancer. Nat Rev Clin Oncol.

[29] Zbili M, Rama S, Benitez M-J, Fronzaroli-Molinieres L, Bialowas A, Boumedine-Guignon N, Garrido JJ, Debanne D (2021). Homeostatic regulation of axonal Kv1.1 channels accounts for both synaptic and intrinsic modifications in the hippocampal CA3 circuit. Proc Natl Acad Sci U S A.

[30] Morgan PJ, Bourboulou R, Filippi C, Koenig-Gambini J, Epsztein J (2019). Kv1.1 contributes to a rapid homeostatic plasticity of intrinsic excitability in CA1 pyramidal neurons in vivo. Elife.

[31] Paulhus K, Ammerman L, Glasscock E (2020). Clinical spectrum of KCNA1 mutations: new insights into episodic ataxia and epilepsy comorbidity. Int J Mol Sci.

[32] Colasante G, Qiu Y, Massimino L, Di Berardino C, Cornford JH, Snowball A, Weston M, Jones SP, Giannelli S, Lieb A (2020). In vivo CRISPRa decreases seizures and rescues cognitive deficits in a rodent model of epilepsy. Brain.

[33] Extrémet J, El Far O, Ankri N, Irani SR, Debanne D, Russier M (2022). An epitope-specific LGI1-autoantibody enhances neuronal excitability by modulating Kv1.1 channel. Cells.

[34] Hatcher A, Yu K, Meyer J, Aiba I, Deneen B, Noebels JL (2020). Pathogenesis of peritumoral hyperexcitability in an immunocompetent CRISPR-based glioblastoma model. J Clin Invest.

[35] D'Adamo MC, Liantonio A, Rolland J-F, Pessia M, Imbrici P (2020). Kv11 channelopathies: pathophysiological mechanisms and therapeutic approaches. Int J Mol Sci.

[36] Trosclair K, Dhaibar HA, Gautier NM, Mishra V, Glasscock E (2020). Neuron-specific Kv1.1 deficiency is sufficient to cause epilepsy, premature death, and cardiorespiratory dysregulation. Neurobiol Dis.

[37] Verdura E, Fons C, Schlüter A, Ruiz M, Fourcade S, Casasnovas C, Castellano A, Pujol A (2020). Complete loss of KCNA1 activity causes neonatal epileptic encephalopathy and dyskinesia. J Med Genet.

[38] Dixon SJ, Lemberg KM, Lamprecht MR, Skouta R, Zaitsev EM, Gleason CE, Patel DN, Bauer AJ, Cantley AM, Yang WS (2012). Ferroptosis: an iron-dependent form of nonapoptotic cell death. Cell.

[39] Zhao L, Zhou X, Xie F, Zhang L, Yan H, Huang J, Zhang C, Zhou F, Chen J, Zhang L (2022). Ferroptosis in cancer and cancer immunotherapy. Cancer Commun.

[40] Wang H, Cheng Y, Mao C, Liu S, Xiao D, Huang J, Tao Y (2021). Emerging mechanisms and targeted therapy of ferroptosis in cancer. Mol Ther.

[41] Lei G, Mao C, Yan Y, Zhuang L, Gan B (2021). Ferroptosis, radiotherapy, and combination therapeutic strategies. Protein Cell.

[42] Lei G, Zhang Y, Koppula P, Liu X, Zhang J, Lin SH, Ajani JA, Xiao Q, Liao Z, Wang H (2020). The role of ferroptosis in ionizing radiation-induced cell death and tumor suppression. Cell Res.

[43] Gan B (2021). Mitochondrial regulation of ferroptosis. J Cell Biol.

[44] Chen X, Li J, Kang R, Klionsky DJ, Tang D (2021). Ferroptosis: machinery and regulation. Autophagy.

[45] Badgley MA, Kremer DM, Maurer HC, DelGiorno KE, Lee H-J, Purohit V, Sagalovskiy IR, Ma A, Kapilian J, Firl CEM (2020). Cysteine depletion induces pancreatic tumor ferroptosis in mice. Science.

[46] Seibt TM, Proneth B, Conrad M (2019). Role of GPX4 in ferroptosis and its pharmacological implication. Free Radic Biol Med.

[47] Zheng X, Wang Q, Zhou Y, Zhang D, Geng Y, Hu W, Wu C, Shi Y, Jiang J (2022). N-acetyltransferase 10 promotes colon cancer progression by inhibiting ferroptosis through N4-acetylation and stabilization of ferroptosis suppressor protein 1 (FSP1) mRNA. Cancer Commun.

[48] Han L, Zhou J, Li L, Wu X, Shi Y, Cui W, Zhang S, Hu Q, Wang J, Bai H (2022). SLC1A5 enhances malignant phenotypes through modulating ferroptosis status and immune microenvironment in glioma. Cell Death Dis.

[49] Zhang D, Man D, Lu J, Jiang Y, Ding B, Su R, Tong R, Chen J, Yang B, Zheng S (2023). Mitochondrial TSPO promotes hepatocellular carcinoma progression through ferroptosis inhibition and immune evasion. Adv Sci.

[50] Li D, Wang Y, Dong C, Chen T, Dong A, Ren J, Li W, Shu G, Yang J, Shen W (2023). CST1 inhibits ferroptosis and promotes gastric cancer metastasis by regulating GPX4 protein stability via OTUB1. Oncogene.

[51] Ebrahimi N, Adelian S, Shakerian S, Afshinpour M, Chaleshtori SR, Rostami N, Rezaei-Tazangi F, Beiranvand S, Hamblin MR, Aref AR (2022). Crosstalk between ferroptosis and the epithelial-mesenchymal transition: Implications for inflammation and cancer therapy. Cytokine Growth Factor Rev.

[52] Müller S, Sindikubwabo F, Cañeque T, Lafon A, Versini A, Lombard B, Loew D, Wu T-D, Ginestier C, Charafe-Jauffret E (2020). CD44 regulates epigenetic plasticity by mediating iron endocytosis. Nat Chem.

[53] You JH, Lee J, Roh J-L (2021). Mitochondrial pyruvate carrier 1 regulates ferroptosis in drug-tolerant persister head and neck cancer cells via epithelial-mesenchymal transition. Cancer Lett.

[54] Wu J, Minikes AM, Gao M, Bian H, Li Y, Stockwell BR, Chen Z-N, Jiang X (2019). Intercellular interaction dictates cancer cell ferroptosis via NF2-YAP signalling. Nature.

[55] Yang W-H, Ding C-KC, Sun T, Rupprecht G, Lin C-C, Hsu D, Chi J-T (2019). The hippo pathway effector TAZ regulates ferroptosis in renal cell carcinoma. Cell reports.

