# Supplementary material for: KCNA1 promotes the growth and invasion of glioblastoma cells through ferroptosis inhibition via upregulating SLC7A11
Source: Cancer Cell Int. 2024 Jan 3;24:7. doi: 10.1186/s12935-023-03199-9 (PMC10765868; doi:10.1186/s12935-023-03199-9)
Supplement: Supplementary file 1 — Additional file 1: Figure S1. H&E staining of GBM tissue. A H&E staining of GBM tissue (the middle and rest scale bar are 500 μm and 100 μm, respectively). [file 12935_2023_3199_MOESM1_ESM.docx]

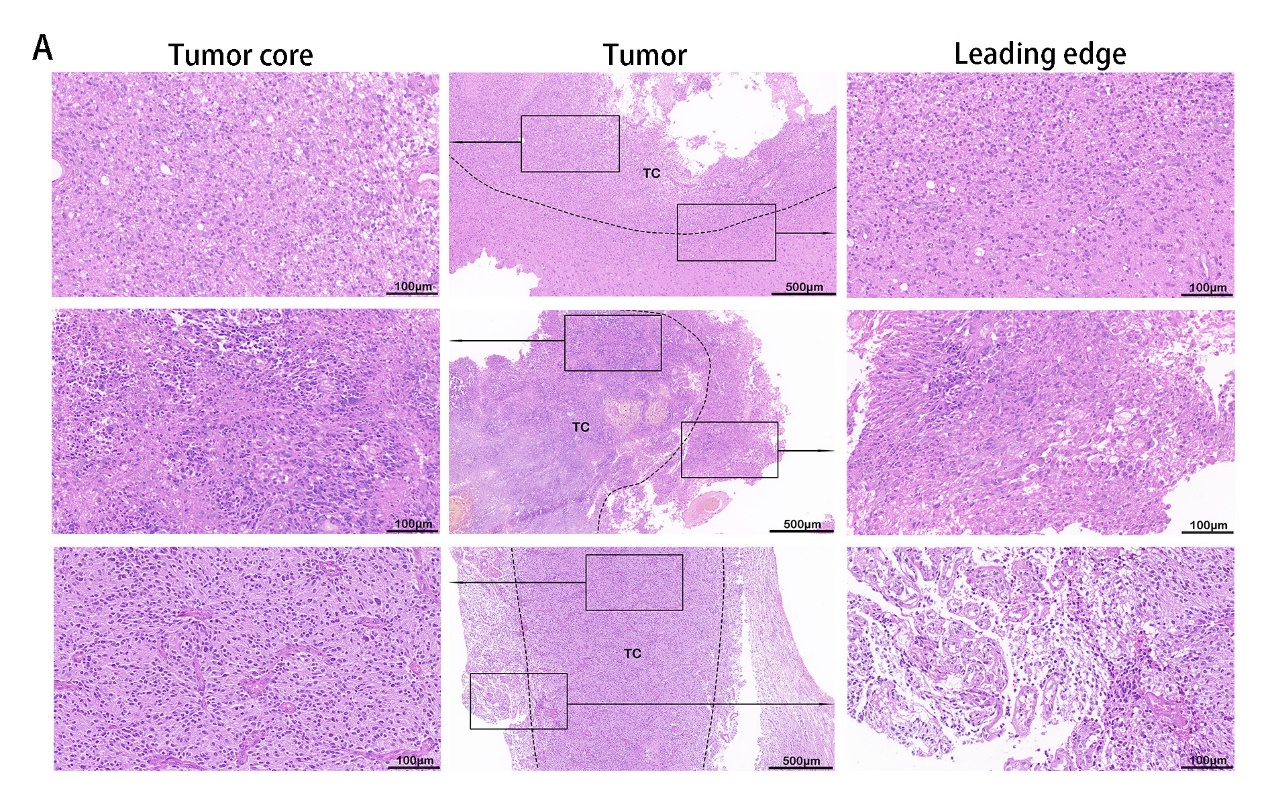


**Fig. S1** H&E staining of GBM tissue. (A) H&E staining of GBM tissue (the middle and rest scale bar are 500 μm and 100 μm, respectively)
