# Supplementary material for: KCNA1 promotes the growth and invasion of glioblastoma cells through ferroptosis inhibition via upregulating SLC7A11
Source: Cancer Cell Int. 2024 Jan 3;24:7. doi: 10.1186/s12935-023-03199-9 (PMC10765868; doi:10.1186/s12935-023-03199-9)
Supplement: Supplementary file 2 — Additional file 2: Figure S2. Knockdown of KCNA1 promotes apoptosis and inhibits invasion in GBM cell lines. A Apoptosis analysis of SHG140 and U87 cells. B scratch assay of SHG140 and U87 cells (Scale bar = 1 mm) C Organoids assay after transfection (Scale bar = 500 μm). One-way ANOVA for multi-group comparisons. n = 3, *p < 0.05, ***p < 0.001, ****p < 0.0 001. [file 12935_2023_3199_MOESM2_ESM.docx]

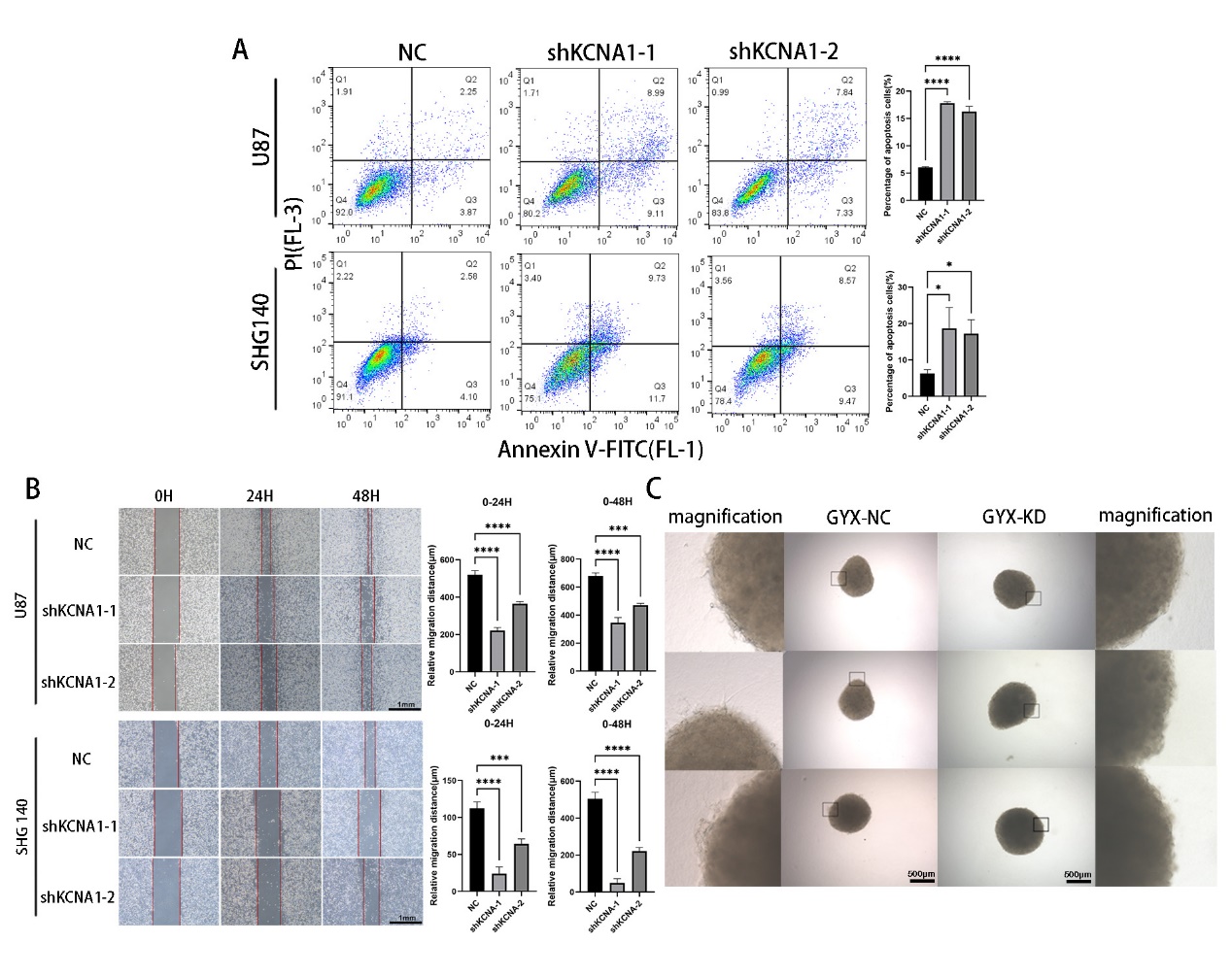


**Fig. S2** Knockdown of KCNA1 promotes apoptosis and inhibits invasion in GBM cell lines. (A) Apoptosis analysis of SHG140 and U87 cells. (B) scratch assay of SHG140 and U87 cells (Scale bar = 1 mm) (C) Organoids assay after transfection (Scale bar = 500 μm). One-way ANOVA for multi-group comparisons. n = 3, *p < 0.05, ***p < 0.001, ****p < 0.0001.
