# Supplementary material for: KCNA1 promotes the growth and invasion of glioblastoma cells through ferroptosis inhibition via upregulating SLC7A11
Source: Cancer Cell Int. 2024 Jan 3;24:7. doi: 10.1186/s12935-023-03199-9 (PMC10765868; doi:10.1186/s12935-023-03199-9)
Supplement: Supplementary file 3 — Additional file 3: Figure S3. Overexpression of KCNA1 inhibits apoptosis and promotes invasion in GBM cell lines. A Apoptosis analysis of SHG140 and U87 cells. B Transwell assay of SHG140 and U87 cells after transfection (Scale bar = 500 μm), C 3D tumor spheroid invasion assay to assess invasion of SHG140 and U87 cells after transfection (Scale bar = 500 μm). Student’s t-test for two-group comparison. n = 3, *p < 0.05, **p < 0.01, ***p < 0.001. [file 12935_2023_3199_MOESM3_ESM.docx]

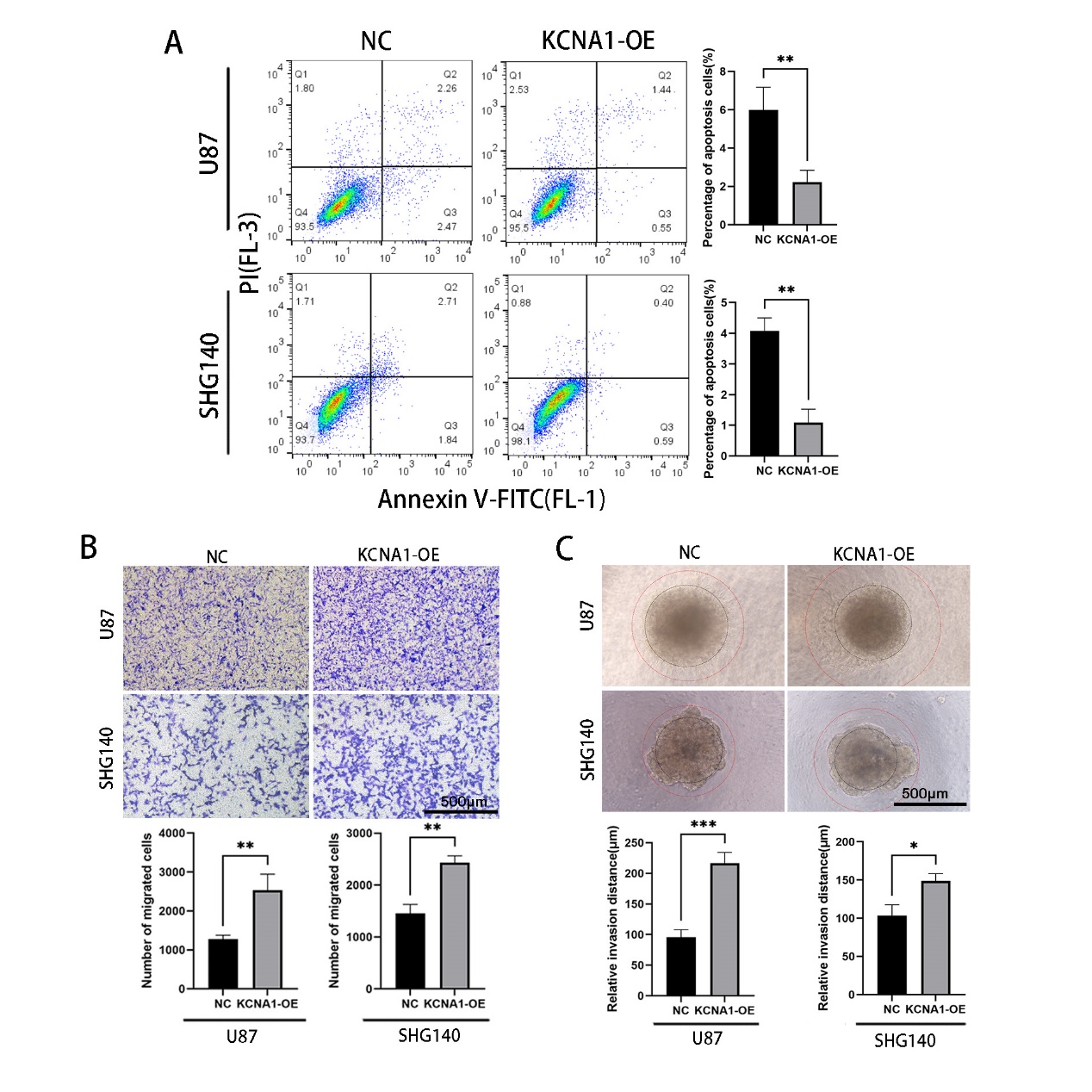


**Fig. S3** Overexpression of KCNA1 inhibits apoptosis and promotes invasion in GBM cell lines. (A) Apoptosis analysis of SHG140 and U87 cells. (B) Transwell assay of SHG140 and U87 cells after transfection (Scale bar = 500 μm), (C) 3D tumor spheroid invasion assay to assess invasion of SHG140 and U87 cells after transfection (Scale bar = 500 μm). Student’s t-test for two-group comparison. n = 3, *p < 0.05, **p < 0.01, ***p < 0.001.
