# Supplementary material for: KCNA1 promotes the growth and invasion of glioblastoma cells through ferroptosis inhibition via upregulating SLC7A11
Source: Cancer Cell Int. 2024 Jan 3;24:7. doi: 10.1186/s12935-023-03199-9 (PMC10765868; doi:10.1186/s12935-023-03199-9)
Supplement: Supplementary file 4 — Additional file 4: Figure S4. Knockdown of SLC7A11 inhibits the migration and invasion of GBM cells. A Scratch assays of U87 and SHG140 cells after transfection (Scale bar = 1 mm), B Transwell assays of U87 and SHG140 cells after transfection (Scale bar = 500 μm). Student’s t-test for two-group comparison. n = 3, *p < 0.05, **p < 0.01, ***p < 0.001. [file 12935_2023_3199_MOESM4_ESM.docx]

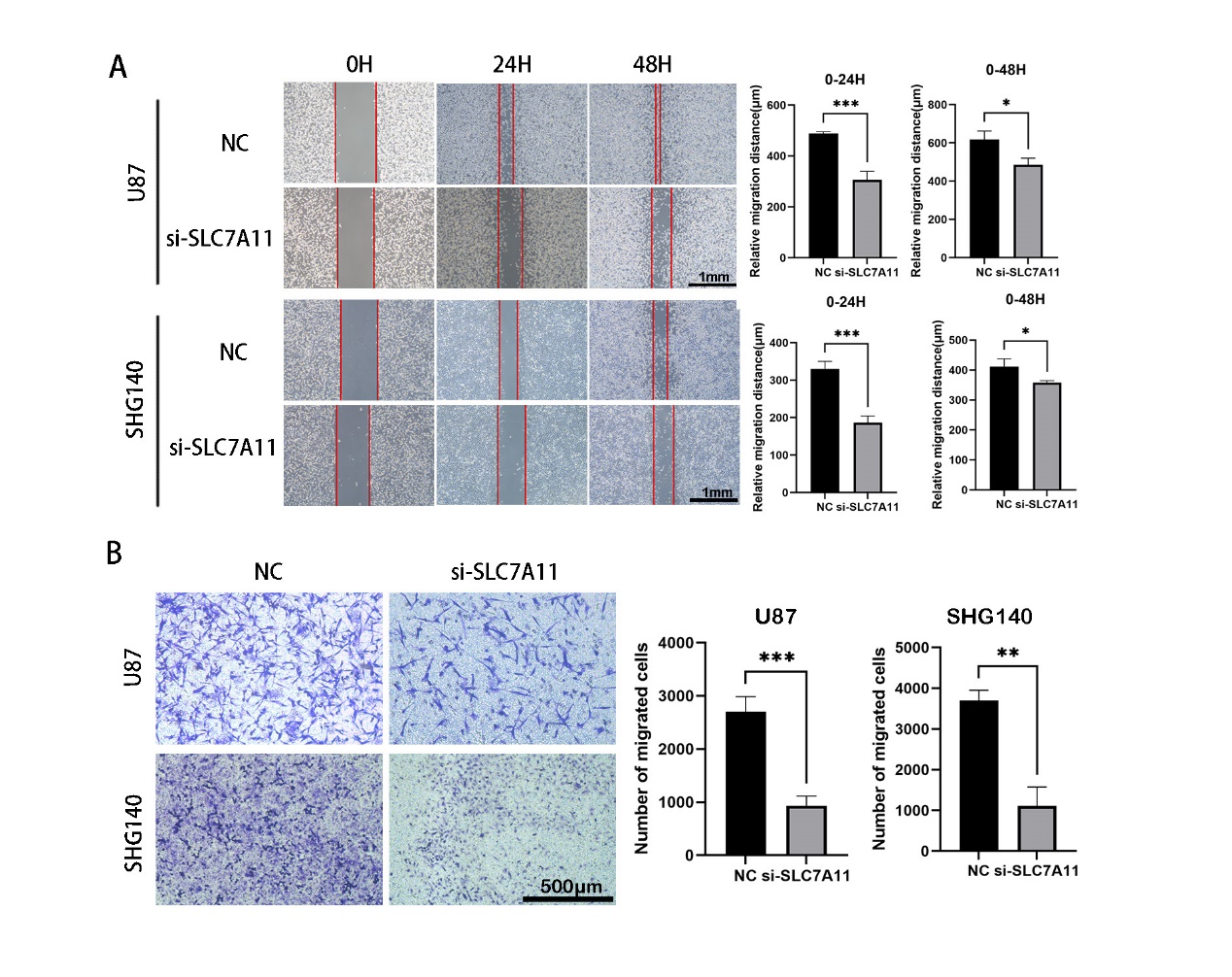


**Fig. S4** Knockdown of SLC7A11 inhibits the migration and invasion of GBM cells. (A) Scratch assays of U87 and SHG140 cells after transfection (Scale bar = 1 mm), (B) Transwell assays of U87 and SHG140 cells after transfection (Scale bar = 500 μm). Student’s t-test for two-group comparison. n = 3, *p < 0.05, **p < 0.01, ***p < 0.001.
